# Frequent Beneficial Mutations during Single-Colony Serial Transfer of *Streptococcus pneumoniae*

**DOI:** 10.1371/journal.pgen.1002232

**Published:** 2011-08-18

**Authors:** Kathleen E. Stevens, Michael E. Sebert

**Affiliations:** 1 Children's Hospital of Philadelphia Research Institute, Philadelphia, Pennsylvania, United States of America; 2 Department of Pediatrics, Children's Hospital of Philadelphia and University of Pennsylvania, Philadelphia, Pennsylvania, United States of America; Université Paris Descartes, INSERM U1001, France

## Abstract

The appearance of new mutations within a population provides the raw material for evolution. The consistent decline in fitness observed in classical mutation accumulation studies has provided support for the long-held view that deleterious mutations are more common than beneficial mutations. Here we present results of a study using a mutation accumulation design with the bacterium *Streptococcus pneumoniae* in which the fitness of the derived populations increased. This rise in fitness was associated specifically with adaptation to survival during brief stationary phase periods between single-colony population bottlenecks. To understand better the population dynamics behind this unanticipated adaptation, we developed a maximum likelihood model describing the processes of mutation and stationary-phase selection in the context of frequent population bottlenecks. Using this model, we estimate that the rate of beneficial mutations may be as high as 4.8×10^−4^ events per genome for each time interval corresponding to the pneumococcal generation time. This rate is several orders of magnitude higher than earlier estimates of beneficial mutation rates in bacteria but supports recent results obtained through the propagation of small populations of *Escherichia coli*. Our findings indicate that beneficial mutations may be relatively frequent in bacteria and suggest that in *S. pneumoniae*, which develops natural competence for transformation, a steady supply of such mutations may be available for sampling by recombination.

## Author Summary

Beneficial mutations have long been considered extremely rare events and were thought to occur with a frequency of approximately one out of a billion times that a bacterium replicates its genome. Rare beneficial mutations would then be amplified by natural selection from the more frequent background of harmful mutations. Mutation accumulation experiments probe the nature of these spontaneous mutations by monitoring changes in fitness of model organisms propagated in the laboratory through numerous generations under conditions where the effects of selection are minimal. Previous mutation accumulation experiments have shown that organisms under study have declined in fitness as random mutations accrue in their genomes, consistent with a predominance of deleterious mutations. We conducted a mutation accumulation study with the bacterial pathogen *S. pneumoniae* in which a broad measure of fitness instead rose. We demonstrate that this unexpected adaptation was due to frequent beneficial mutations that were further amplified by selection in stationary-phase bacterial colonies. Together with recent work using *E. coli*, these results demonstrate that beneficial mutations can be common in bacteria and may contribute to our understanding of the evolution of traits such as antibiotic resistance and virulence.

## Introduction

Spontaneous mutations provide the underlying variability on which selection acts to drive evolution. Among newly-arising mutations that impact fitness, the balance between those that are beneficial and those that are deleterious has appeared to be heavily skewed toward maladaptive changes. This view has been supported by mutation accumulation (MA) studies, in which experimental lines of model organisms, including *Drosophila* [1], [2], *Escherichia coli* [3], [4], *Caenorhabditis elegans* [5], [6], *Arabidopsis thaliana* [7], *Tetrahymena* [8], *Saccharomyces cerevisiae* [9] and *Salmonella typhimurium* [10], have lost fitness when propagated under conditions of relaxed selection. Analysis of such MA lines in *E. coli* yielded an estimate for the rate of deleterious mutations as being at least 1.7×10^−4^ events per genome per generation [3]. In contrast, beneficial mutations were believed to occur in bacterial populations only at frequencies near 10^−8^ to 10^−9^ [11], [12].

More recent evidence, however, indicates that beneficial mutations are far more common in *E. coli* than previously recognized. In large populations of asexual microbes such as bacteria, clonal interference between competing mutations can prevent the fixation of beneficial mutations and limit the overall rate of adaptation [13]. Overcoming the experimental bias imposed by clonal interference by propagating populations of *E. coli* at a small effective population size (N_e_), Perfeito *et al.* recently demonstrated in this model organism that beneficial mutations occurred as frequently as 2×10^−5^ per genome per generation [14].

We address here the frequency of beneficial mutations in the gram-positive bacterium, *Streptococcus pneumoniae*. This organism is a common pathogen of the human respiratory tract for which questions of the acquisition and spread of beneficial mutations have taken on additional medical importance as the bacterium has developed increasing resistance to antibiotics [15] and has shifted its population structure in response to the pressure of vaccination [16]. In contrast to *E. coli*, *S. pneumoniae* is a naturally-transformable bacterium in which recombination reduces the clonality of the population [17], [18] and may thereby facilitate more rapid adaptation. We present unexpected results from an MA experiment with *S. pneumoniae* in which beneficial mutations were sufficiently frequent to cause—in combination with selection in stationary-phase colonies—a net increase in fitness of the propagated populations. These findings support the emerging conclusion that beneficial mutations may be relatively frequent in bacteria.

## Results

### Increasing Fitness during Serial Transfer of Single Pneumococcal Colonies

**Figure 1:**
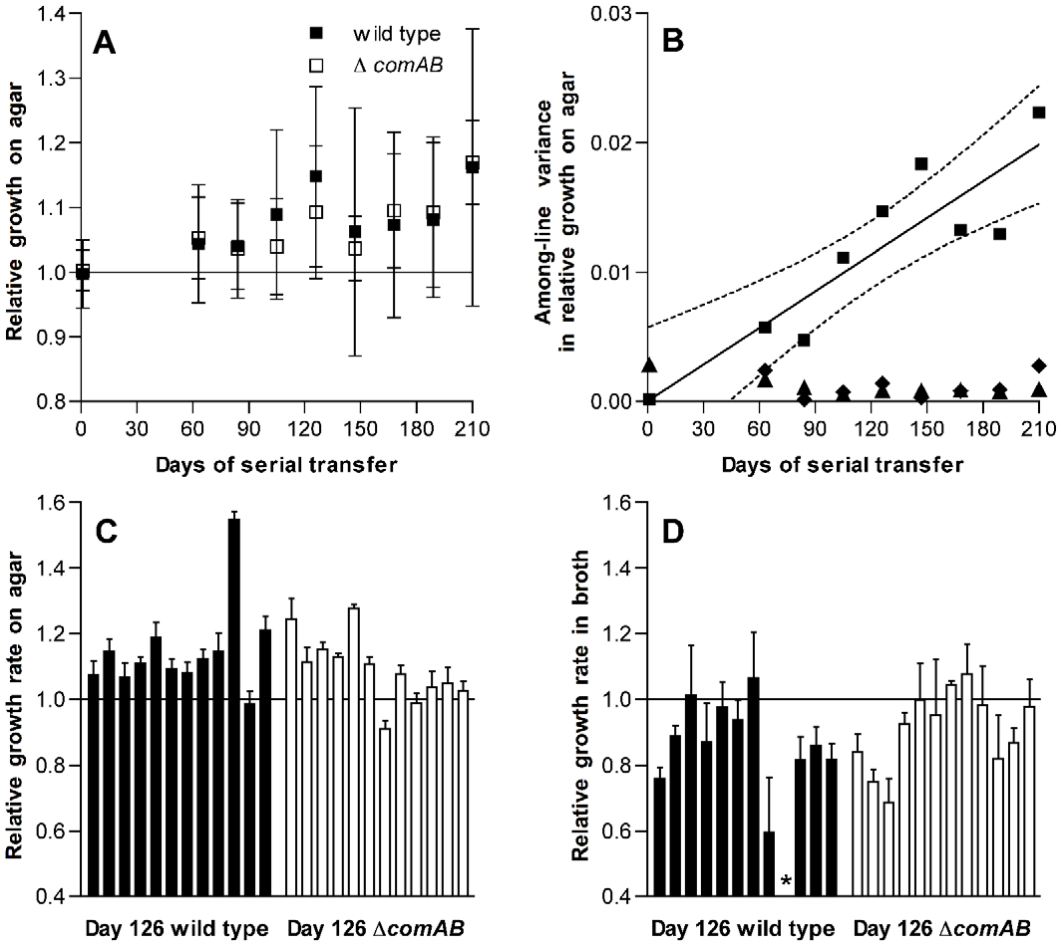
Changes in fitness of *S. pneumoniae* over single-colony serial transfer. (A) Relative growth after 24 h of incubation on THY agar plates expressed as the ratio of log_2_(CFU/colony) for passaged isolates based on 3 replicate measurements for each isolate compared to day 1 control isolates. Symbols represent average fitness values for independently passaged lines ± S.D. The increase in fitness with duration of passage is significant (*F*_[8,185]_ = 3.65, *P* = 0.0006), but differences between Δ*comAB* and wild-type lines were not significant (*F*_[1,185]_ = 0.18, *P* = 0.67). (B) Variance components of fitness measurements based on relative log_2_(CFU/colony) for serial transfer lines. Among-line variances for passaged strains are shown with squares. Linear regression of the among-line variance against duration of passage is shown by a solid line, with dotted lines indicating 95% confidence intervals. Among-line variances for day 1 control isolates assayed in parallel at each stage of serial transfer are shown with diamonds. Triangles represent within-line variances for passaged strains. Data from wild-type and Δ*comAB* lines are pooled. (C) Relative growth on agar plates, as in panel A, for individual isolates after 126 days of serial transfer. The effect of lineage on fitness was significant (*F*_[23,71]_ = 34.01, *P*<0.0001). n = 3 for each lineage. (D) Relative growth in THY broth, expressed as the ratio of maximum growth rates for passaged isolates compared to day 1 control isolates, after 126 days of serial transfer. The effect of lineage on fitness was significant (*F*_[22,68]_ = 4.96, *P*<0.0001). n = 3. Bars represent mean values ± S.D. The asterisk indicates an isolate that was unable to grow in broth culture.

Initially intending to characterize the rate of accumulation of deleterious mutations in *S. pneumoniae*, we designed an in vitro serial transfer experiment using a classical mutation accumulation design under conditions of weak selection and strong genetic drift. Forty bacterial lines were passaged in parallel over a period of 210 days by transferring single colonies each day to fresh THY agar plates. We anticipated a general decline in fitness among these lines due to accumulation of deleterious mutations in the setting of frequent population bottlenecks, as has been previously described in studies with *Escherichia coli* and *Salmonella* [3], [10]. Unanticipated results described below, however, suggested that adaptation had occurred despite these conditions and caused us to revise our plans so as to investigate this adaptation in detail. Evidence for adaptation emerged when, intending to exclude the possibility of specific adaptation to the conditions of growth on THY agar plates during passage, we tested a sample consisting of 24 consecutive lines out of the 40 that had been passaged. Fitness for growth on the agar surface was estimated by dissecting individual colonies from plates after 24 h of incubation and measuring the number of colony-forming units (CFU) in each mature colony. As described below, the 24 lines tested consisted of 12 lines each in wild-type and competence-deficient Δ*comAB* backgrounds. Unexpectedly, the fitness of these bacterial lines—as measured by log_2_(CFU/colony) values—generally rose as the experiment progressed (Figure 1A and 1C). Because increasing fitness did not match the predictions of the mutation accumulation model under which the experiment had been planned, the scope of the project was revised at this point to focus on characterizing the apparent adaptation seen among these 24 serial transfer lines that had been initially tested.

Because pneumococcus is a naturally-transformable bacterium [19], [20] and we had initially been interested in later testing whether horizontal gene transfer between populations that have independently accumulated deleterious mutations can restore fitness, half of these lines had been initiated from a wild-type progenitor whereas the other half were derived from an isogenic, competence-deficient Δ*comAB* mutant. In comparing the wild-type and Δ*comAB* lines (12 lines of each genotype), we did not expect substantial differences between the groups because the THY medium used during this experiment is not permissive for development of spontaneous competence when bacteria are grown in broth culture and because daily serial transfer was expected to limit the time available for selection of recombinants after a transformation event. We could not, however, exclude the possibility that a fraction of the bacteria within a colony might develop competence at some point during the growth cycle and therefore examined our data for evidence of differences between wild-type and Δ*comAB* lines. No significant differences in fitness were observed between the groups (Figure 1A), and data from these two sets of lines were pooled for subsequent analyses.

Among-line variance in fitness also increased over the course of serial transfer, consistent with mutational divergence of the passaged lines (Figure 1B). In contrast, within-line variance remained low throughout the experiment. Likewise, the among-line variance measured for the day 1 reference strains, which were analyzed in parallel with the evolved strains at each time point, did not increase as the experiment progressed.

**Figure 2:**
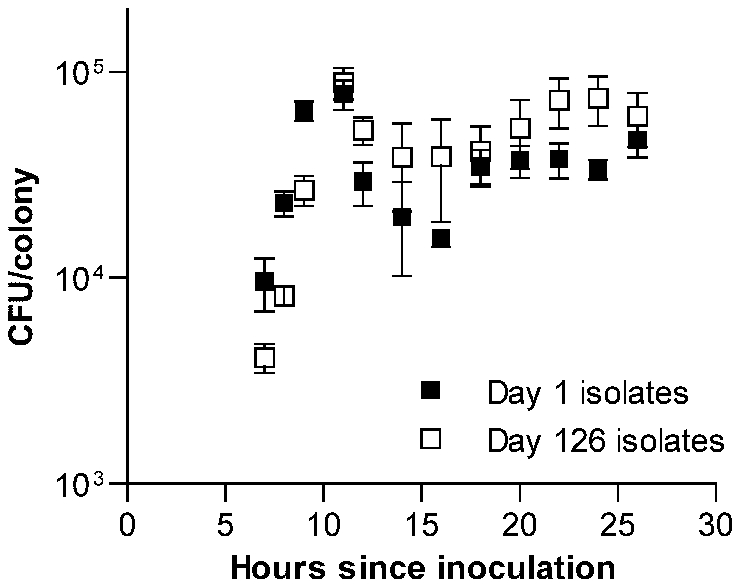
Time series examination of the development of *S. pneumoniae* colonies. Average CFU/colony values ± S.E.M. are shown for 6 serial transfer lines after 7 to 26 h of incubation on THY agar plates. Values are higher during exponential phase (7 to 11 h) for day 1 isolates (*F*[_1, 40_] = 4.51, *P* = 0.04) but higher during stationary phase (12 to 26 h) for day 126 isolates (*F*_[1,80]_ = 11.31, *P* = 0.0012).

To determine whether this increase in fitness was specific to the context of bacterial growth on the surface of agar plates, we next measured the growth of each isolate in THY broth cultures and estimated fitness in this setting based on the maximum rate of growth. In contrast to CFU/colony measurements (Figure 1C), growth rates in broth culture for many lines declined over the first 126 days of serial transfer (Figure 1D). The passaged isolate from one wild-type line even appeared to have lost the ability to grow in broth culture altogether. The adaptation of these pneumococcal lines therefore appeared to be specific for survival on agar plates and was further characterized by dissecting colonies off plates at intervals ranging from 7 to 26 h after inoculation. These time-series measurements of bacterial CFU/colony were performed for a sample consisting of the first 6 lines in the collection (representing 3 wild-type and 3 Δ*comAB* lines, Figure 2) using isolates taken at days 1 and 126 of serial transfer. Developing colonies displayed an initial phase of active growth that extended through the first 11 h following inoculation. During this phase, growth of the colonies was approximately exponential. In this period, growth of the day 126 isolates lagged behind that of the day 1 isolates, consistent with the lower maximal rate of growth seen in the broth assays for the former samples.

After the initial phase of exponential growth, colonies entered what we refer to here broadly as stationary phase. Although changes in CFU/colony values observed during this period indicate that bacterial replication and death continue to at least some extent, this phase is characterized by birth and death rates being approximately equal. Within this stage, viable bacterial counts in maturing colonies briefly declined, followed by a slow rebound and plateau. It was during this stationary phase of colony development that the advantage of the passaged isolates became evident. When data were examined for each line individually, one line (shown as line B in Figure S1A and S1B) was found to have acquired a markedly greater stationary phase advantage than was seen in the other five lines. Even when this line was excluded, however, a pattern indicating adaptation to survival in stationary phase was seen among the remaining evolved isolates (Figure S1C). Notably, this advantage was particularly prominent from 22 to 24 h after inoculation, matching the time interval at which colonies were picked for serial transfer.

Although the definition of fitness is complicated in this serial transfer model by repeated exposure to both exponential and stationary phase growth conditions, it is important to note that the CFU/colony value at the end of a 24 h colony growth cycle reflects the net result of events during both the exponential and stationary phases of colony development. In this regard, CFU/colony measurements made at the end of the 24 h colony growth cycle reflect overall fitness of the passaged lines under the conditions of experimental propagation. Increases in CFU/colony observed over the course of the experiment—while driven by improved survival during stationary phase—therefore indicate increased overall fitness of the passaged lines. Because our serial transfer model did not permit seeding of individual colonies from a mixed inoculum, CFU/colony values provide a proxy for absolute fitness of strains in isolation but do not directly measure relative fitness. Potential implications of differences between relative and absolute fitness, and particularly the possibility of frequency-dependent variation in the strength of selection, are considered later.

### Pleiotropic Effects of Accumulating Mutations

**Figure 3:**
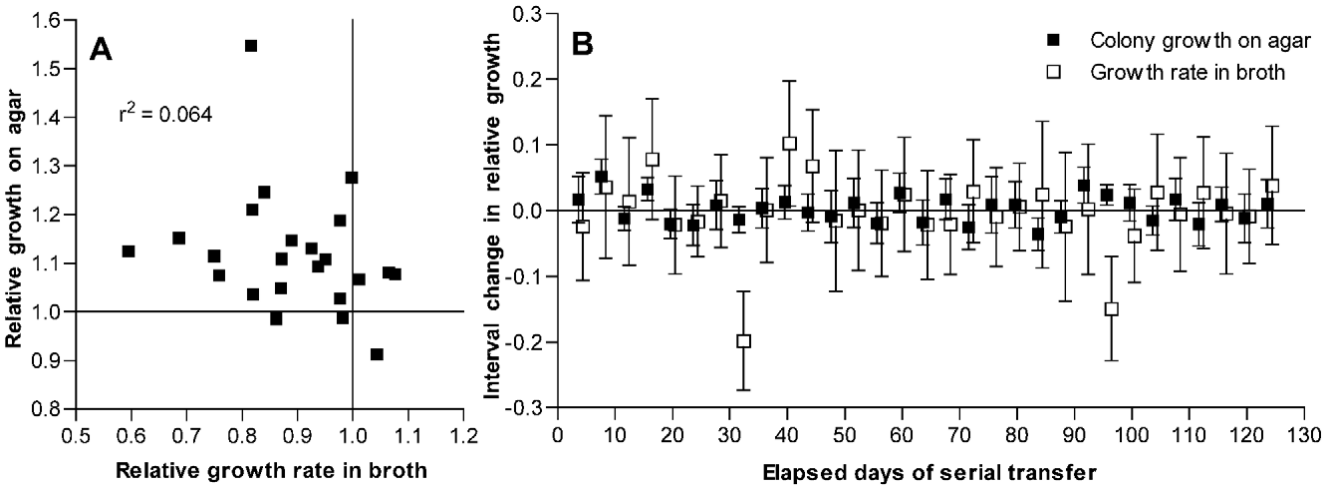
Changes in fitness of passaged isolates for growth on agar plates as compared to broth. (A) Growth on THY agar plates as compared to growth in THY broth for isolates after 126 days of serial transfer. Values are given relative to the average of day 1 control isolates as described for Figure 1. Correlation between these measurements is not significant (*P* = 0.24). (B) Interval changes in relative growth on THY agar plates or in THY broth culture for isolates from a single *S. pneumoniae* line taken every 4 days during serial transfer. Growth is measured as log_2_(CFU/colony) or as maximum broth growth rate, both relative to similar measurements for the preceding sample in the series. Error bars represent 95% confidence intervals for the differences between consecutive samples from individual t-tests based on at least 10 measurements of broth growth and 12 measurements of CFU/colony for each isolate. ANOVA with Bonferroni correction demonstrated significant changes in broth fitness at days 32 and 96 (*P*<0.001 for each post test) and in agar fitness at days 8 (*P*<0.001) and 92 (*P*<0.05). Additional CFU/colony measurements were performed for isolates at days 16 and 96 that showed possible changes on initial testing. By individual t-tests, these changes at day 16 (*P* = 0.0005, n = 24) and day 96 (*P* = 0.0034, n = 51) were also significant.

Because mutations that are advantageous during stationary phase may be deleterious under conditions of rapid growth [21], we considered whether the decline in broth culture growth rates observed with passage might result in part from pleiotropic effects of mutations that were adaptive in the context of colonies grown on an agar plate. No correlation was observed between these two fitness measurements among the isolates that had been passaged for 126 days (Figure 3A). However, because some of these isolates may have accumulated multiple mutations and because the magnitude of an adaptive benefit may not correlate with the magnitude of a linked pleiotropic effect, the absence of such a correlation among the day 126 isolates cannot exclude the possibility that antagonistic pleiotropy contributes to the observed fitness changes.

To examine the relationship between these fitness changes more closely, a single bacterial line (shown in Figure S1 as line F, which had a Δ*comAB* genotype) was chosen at random for further testing from among several lines for which additional samples had been frozen every 4 days during serial transfer. For this analysis, line B—which had shown distinctly stronger adaptation (Figure S1B)—was excluded from consideration as a potential outlier. Assuming that only rarely would more than one fitness-modifying mutation become fixed during a short interval, these samples served to isolate largely the effects of individual mutations. For each isolate in this series, the change in fitness as compared to that of the preceding isolate was measured in terms of both CFU/colony values and maximal broth growth rates (Figure 3B). Between most pairs of consecutive isolates, no significant change in either value was observed. At four time points, however, significant increases in fitness for growth on agar plates were observed, and at one of these time points (day 96) a significant decline in the growth rate in broth culture was seen to accompany this change. However, not all mutations that were beneficial during growth on plates were deleterious in broth, and one mutational event was observed that appeared to be deleterious for growth in broth without conferring a benefit for growth on agar plates (day 32). Occasional fixation of such a strictly deleterious mutation is in keeping with the original experimental design promoting mutation accumulation. Nonetheless, the observation of inversely correlated changes in broth and agar fitness measurements at day 96 also suggests that a component of the decrease in maximum growth rates seen in broth culture resulted from pleiotropic effects of mutations that were beneficial for growth on solid media rather than from the accumulation of strictly deleterious mutations. Consequently, the decline in growth rates of these samples in broth cannot be used here to estimate the rate of deleterious mutations in *S. pneumoniae* because it is likely to be confounded by the effects of beneficial mutations that are selected during growth on agar but are pleiotropically deleterious in broth.

### Estimation of the Beneficial Mutation Rate in *S. pneumoniae*

Previous studies of the acquisition and selection of beneficial mutations in bacteria have typically been conducted using larger populations and without explicit consideration of the role of events during stationary phase [22]–[24]. While the development of adaptive mutations during single, prolonged episodes of stationary phase survival has been an issue of controversy [25]–[27], the evolution of bacterial populations experiencing repeated exposure to brief stationary phase conditions has not to our knowledge been explored systematically. Under these conditions in which the maximum population size within a colony remains under 10^5^ and daily single-colony bottlenecks limit the duration of selection, multiple beneficial mutations should be unlikely to arise and become amplified to a level sufficient to compete with each other during a single colony growth cycle. The adaptation observed in this system therefore offers an opportunity to measure beneficial mutations largely free from clonal interference between competing mutations that can bias estimates of the mutation rate downwards (reviewed in [13]).

In order to understand the conditions that led to increased fitness for stationary phase survival during our serial transfer experiment, we developed a maximum likelihood (ML) model describing the processes of mutation and stationary phase selection in populations subjected to the strong drift imposed by serial transfer of single colonies. This model was used to estimate the rate of beneficial mutations and the distribution of effect strengths of such mutations. Parameters were evaluated by comparing output of the simulation model with fitness values—as measured by growth on agar plates—from our experimental serial transfer lines after 126 days of passage. This time point was selected because average fitness had shown a generally consistent and increasing trend over these first 126 days. The model assumed a constant beneficial mutation rate and effect distribution over time without epistasis, and these assumptions appeared more likely to be satisfied during this initial phase of the experiment than later when the gain in fitness was less steady.

**Figure 4:**
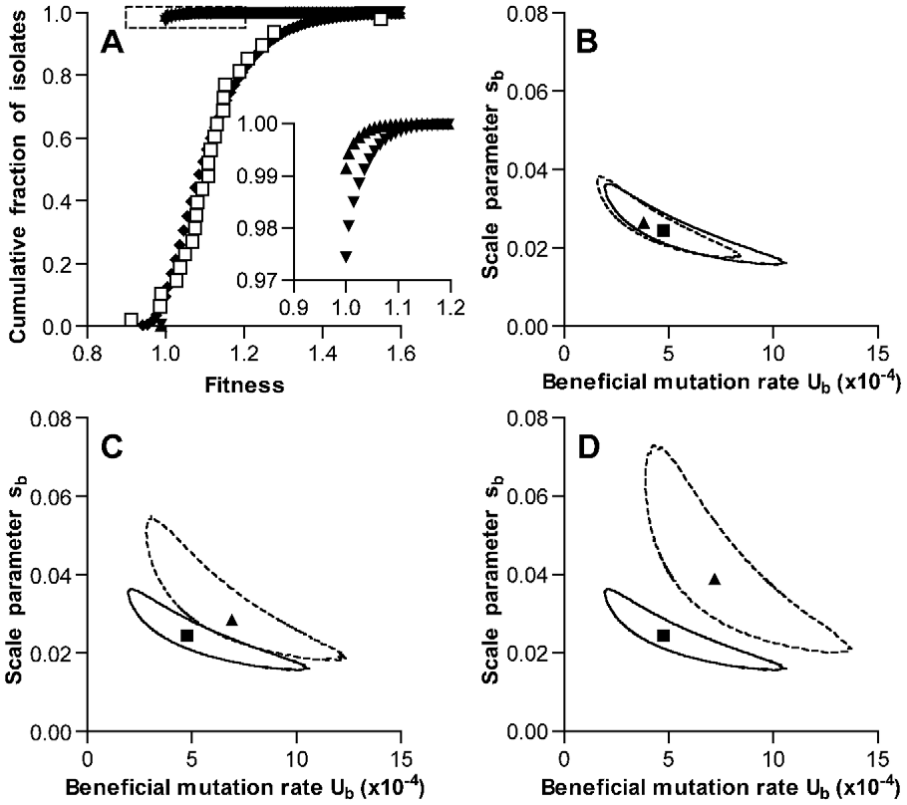
Simulation of fitness changes during single-colony serial transfer of *S. pneumoniae*. (A) The cumulative distribution of fitness values predicted by the ML model as described in the text is shown following the exponential phase of a single growth cycle (mutation only, triangles), after stationary phase of a single growth cycle (further mutation plus selection, inverted triangles), and after iteration of 126 growth cycles (diamonds). Values measured for experimental lines are shown as open squares for comparison. The inset expands the portion of the graph indicated by the dotted lines to show the frequencies of beneficial mutations within a single growth cycle. Parameters for the simulation shown were *U*_b_ = 4.8×10^−4^, s_b_ = 0.025, *U*_d_ = 1.7×10^−4^, and s_d_ = 0.012. Values for *U*_b_ and s_b_ represent ML estimates. Selection was permitted throughout stationary phase, and mutation rates were held constant during the growth cycle. (B–D) ML estimates for *U*_b_ and s_b_ derived under different model assumptions. Results obtained for the set of conditions used in A, while allowing *U*_b_ and s_b_ to vary, are shown in each panel for comparison with a square symbol (ML estimate) and solid line (95% confidence contour). The effects of varying a single model condition on ML values and 95% confidence contours are shown with triangles and dotted lines, respectively. Alternative model conditions were (B) no deleterious mutations, (C) selection limited to the final 6 time intervals of stationary phase, and (D) no selection.

Details of the model are presented in Materials and Methods. Because average fitness increased during the experiment and only 1 line showed decreased fitness, our data did not permit independent estimates of the deleterious mutation rate *U*_d_ or the change in fitness s_d_ associated with such mutations. As these values have not been determined empirically for *S. pneumoniae*, parameters for the model were chosen based on a mutation accumulation study conducted in *E. coli* that had reported *U*_d_ of 1.7×10^−4^ per genome per generation and s_d_ of 0.012 [3]. Under these conditions and allowing for selection throughout stationary phase, the ML estimate for the beneficial mutation rate *U*_b_ was 4.8×10^−4^ per genome per time interval (with intervals corresponding to the 40 min generation time of the bacterium during exponential growth, Figure 4B). The corresponding ML estimate for the scale parameter s_b_ describing an exponential distribution of fitness effects of these mutations was 0.025. (Note that s_b_ also represents the mean effect of beneficial mutations.) The predicted fitness distribution for simulated bacterial lines after 126 growth cycles using these parameters matched the values measured in our serial transfer experiment well (Figure 4A). Most notably, this analysis indicated that the beneficial mutation rate in *S. pneumoniae* may be particularly high.

We then tested the impact of the specific deleterious mutation parameters used in the model by generating new estimates for *U*_b_ and s_b_ under the extreme case in which deleterious mutations were not permitted. This change did not substantially affect the output of the model (ML estimates for *U*_b_ = 3.8×10^−4^ and for s_b_ = 0.027, Figure 4B). We cannot exclude the possibility that deleterious mutations may on the other hand be more frequent or more severe in *S. pneumoniae* as compared to *E. coli*. In that case, the beneficial mutation rate calculated here would likely underestimate the actual rate. One variation that was explored in considering the possibility of a higher deleterious mutation rate was a provisional estimate based on Bateman-Mukai [28], [29] analysis of the reduction in broth growth rates seen with our experimental lines. As noted earlier, however, this estimate appears to be biased by the pleiotropic effects of mutations selected during stationary phase and is not suggested to represent an accurate measurement of the deleterious mutation rate in *S. pneumoniae*. Nonetheless, such analysis would have generated an estimate for the deleterious mutation rate *U*_min_ of 2.5×10^−4^ events per genome per 40 min time interval. This represents a point estimate based on the magnitude and variance of fitness changes seen among our lines between days 1 and 126. If deleterious mutations occurred only during the 16 generations per growth cycle minimally required to generate colonies having the population size observed after exponential phase growth, the Bateman-Mukai estimate for our experiment would increase to 5.7×10^−4^ events per genome per generation. We therefore simulated the serial transfer process using this higher value for *U*_d_ of 5.7×10^−4^. As anticipated, the increase in *U*_d_ raised the ML estimate for the beneficial mutation rate (*U*_b_ = 7.5×10^−4^ and for s_b_ = 0.021, Figure S2A).

The effect of our assumption regarding the extent of the colony growth cycle during which stationary phase selection is effective was also explored by varying this structural parameter of the model. ML estimates were generated under an alternative model in which selection was permitted only during the last 4 hours (6 time intervals) of the growth cycle. This final period, when competition for depleted nutrients may be most severe, was the stage at which our experimental passaged isolates showed the most consistent advantage (Figure 2 and Figure S1C). Under these conditions of limited selection, ML values for *U*_b_ (6.9×10^−4^) and s_b_ (0.029) were slightly higher than when selection was allowed throughout stationary phase (Figure 4C). We also explored the limiting condition where stationary phase selection did not occur and mutations only accumulated neutrally (Figure 4D). As expected, this condition generated higher ML values for *U*_b_ (7.2×10^−4^) and s_b_ (0.039). Finally, we tested the impact of the assumption that the mutation rate remained constant during fixed time intervals of both exponential growth and stationary phase survival. When mutations were restricted to occur only during exponential growth, ML estimates for *U*_b_ and s_b_ were 8.4×10^−4^ and 0.024, respectively (Figure S2B). Despite small differences in output values, these model variations all support the presence of a high rate of beneficial mutation in *S. pneumoniae* in the range of 10^−4^ and possibly as high as 10^−3^ per genome per generation time interval.

Considering the potential limitations of the above model in which events within a growth cycle are described deterministically, a wholly stochastic model was also developed describing the replication, mutation and selection of individual bacteria within a single colony at each serial transfer step. Simulation of the serial transfer process by this model was more than 100,000 times slower than with the semi-deterministic model and was therefore not suitable for the comprehensive exploration of parameter space required for maximum likelihood estimation. Nonetheless, the distribution of fitness values after the simulated serial transfer process generated by the entirely stochastic model was similar to that of our experimental data when mutation and selection parameters matching the ML estimates derived from the faster model were used (Figure S3). This similarity demonstrates that the simplifying assumptions required by the semi-deterministic model do not strongly distort its behavior.

## Discussion

Prior mutation accumulation studies in bacteria and eukaryotes have uniformly shown that average fitness of the propagated populations decreases [1]–[3], [5]–[9], [29] even though increased fitness has been observed for individual lines in some studies [4], [30]–[32]. In contrast, the average fitness of *S. pneumoniae* lines in our experiment rose despite a serial transfer protocol of daily single-colony population bottlenecks. Because adaptation among these lines was specific for survival in stationary-phase bacterial colonies, we infer that selection during this period of the growth cycle contributed to the probability of fixation of beneficial mutations. Considering that microbial populations increase to large sizes even when subjected to frequent bottlenecks, assays with these organisms may be particularly prone to unintended selection during brief and recurrent episodes of stationary phase survival. Mutation accumulation systems have been designed to minimize selection, and therefore evidence that selection influences the outcome of a classically-designed microbial mutation accumulation experiment raises questions about the assumptions underlying these experiments.

Detailed examination of a pneumococcal line that was sampled at high frequency during the serial transfer process demonstrated that on at least one occasion the fixation of a mutation adaptive for growth on agar was accompanied by a large decrease in the rate of growth in broth culture. This observation suggests that antagonistic pleiotropy may be responsible for a portion of the overall loss of fitness for growth in broth that was observed with the larger set of evolved strains as they adapted to stationary phase conditions. Such an inverse relationship between fitness for survival during stationary phase and maximal exponential growth rate has been reported previously in *E. coli* [21] and is consistent with the hypothesis of a trade-off between the optimization of self-preservation and nutritional competence (referred to as the SPANC balance) [33], [34].

Although we initially set out to assess the rate of deleterious mutations in *S. pneumoniae*, this antagonistic pleiotropy appears to confound attempts to estimate deleterious mutation parameters based on the declining growth rates observed in broth cultures. It is not known whether the stationary-phase adaptation that appears to be the source of this bias is unique to *S. pneumoniae* or might extend to other bacteria. This bias may be limited if selection during stationary phase is weak. It is noteworthy, however, that the mutation accumulation experiment of Kibota and Lynch in *E. coli* that reported the anticipated decline in fitness exclusively employed fitness assays measuring maximal rates of growth in broth cultures [3]. For comparison with this earlier study, we determined that application of a similar Bateman-Mukai analysis to our data would have generated an estimate for the deleterious mutation rate *U*_min_ of 2.5×10^−4^ events per genome per 40 min time interval. Although this is only a point estimate based on data for two time points, the result is similar to the value of 1.7×10^−4^ per genome per generation derived from the work in *E. coli* [3]. Due to the limitations noted above, however, the actual rate of deleterious mutations in *S. pneumoniae* remains to be determined.

Attempting to understand the process of selection in fluctuating populations, we developed a ML model of mutation and selection in the context of frequent single-colony bottlenecks. This model analyzed the fitness values measured for our experimental lines after 126 days of passage to estimate parameters for beneficial mutations. These fitness values, which were derived from assays of CFU/colony on agar plates, suggested that the lines had attained relatively modest increases in fitness and were consistent with weak selection in the system. (The possibility of stronger, frequency-dependent variation in the strength of selection is considered later.) Our model demonstrated that weak selection even in the setting of frequent single-colony bottlenecks would be sufficient to explain the adaptation that was observed if the beneficial mutation rate, *U*_b_, were in the range of 4.8×10^−4^ per genome per 40 min time interval. Consistent with weak selection in the model, similar ML estimates of *U*_b_ were obtained even when selection was restricted or eliminated (Figure 4C and 4D). The model furthermore predicted that selection extending throughout stationary phase (as in Figure 4A, in which newly arising beneficial mutations have an average selection coefficient of 0.025) would increase the average selection coefficient only to 0.038 for beneficial mutations that become fixed. This estimate is in good agreement with the average selective advantage of 0.037 measured for the 4 beneficial mutational events (seen at days 8,16, 92 and 96 of serial transfer in Figure 3B) that were observed during our high-frequency analysis of one experimental line.

If selection in the serial transfer system were weak, a high beneficial mutation rate would therefore be required as the major force contributing to adaptation among these lines of *S. pneumoniae*. Although oxidative stress from strong endogenous production of peroxide is a major source of mutations in this organism [35], mutation rates in *S. pneumoniae* do not appear to be unusually high compared to other bacteria. The rate of point mutations conferring resistance to the antibiotic optochin, for instance, was estimated by fluctuation analysis to be 1.4×10^−8^ per cell division for the D39 strain used in our study [36]. It is possible, however, that bacteria experience more intense oxidative stress, and consequently a higher mutation rate, at a high density within stationary-phase colonies than during growth in broth. A higher mutation rate has also been suggested to occur in aging colonies of *E. coli* (over a period of 7 days) associated with the induction of an RpoS stress response [37], and a high deleterious mutation rate has been reported for *E. coli* during prolonged episodes of stationary phase (100 days) [38]. While both of these observations have been subject to controversy [26], [39], their relevance to the current study may be reduced by the consideration that *S. pneumoniae* lacks a homologue of the RpoS stationary-phase sigma factor.

Regardless of the overall mutation rate, our results indicate that beneficial mutations in particular may be relatively common for this organism when single colonies are propagated in vitro (as compared to deleterious mutations, which may yet be more frequent but are not amplified by selection). The balance of mutational effects between those that are beneficial and those that are deleterious may shift as the fitness of an organism changes [40], [41]. For phage in which fitness has been substantially degraded through previous mutations, additional random mutations, for instance, have been shown even to cause a net restoration of fitness [42]. In contrast to such phage, however, the D39 isolate of *S. pneumoniae* used for this experiment is a laboratory strain that has not been subjected to mutagenesis and is fit enough that it retains virulence in animal models. To the extent, however, that this organism is better adapted to exponential growth in broth culture than to survival within a stationary-phase colony, this consideration may have contributed to the net increase in fitness we observed for colony growth.

An alternative to the above scenario of weak selection is the possibility of strong frequency-dependent variation in the strength of selection within developing bacterial colonies. If beneficial mutations generate a GASP (growth advantage in stationary phase) phenotype [43] that allows mutants to continue growing after wild-type bacteria in a colony have stopped replicating, the initial gain in relative fitness associated with such mutations may potentially be large. A newly arisen mutant might increase from a single individual to a sizeable percentage of the total colony population within a single growth cycle. Yet once a GASP mutant becomes common in the population, its relative fitness advantage would decline because the higher carrying capacity associated with the mutation would be reached after fewer generations of bacterial growth. When fitness of an evolved strain is measured in isolation, such a GASP mutation might confer only a small fitness benefit—as measured by CFU/colony—reflecting a modest increase in the carrying capacity of the colony. If such frequency-dependent selection of GASP mutants was a source of strong selection in our serial transfer lines, the rate of beneficial mutations may be lower than we have estimated. Because we are unable to initiate individual pneumococcal colonies from a mixed inoculum for competitive fitness assays, we have not been able to evaluate this possibility directly.

The potential effects of recurrent exposure to stationary phase and frequency-dependent variation in the strength of selection on other microbial systems of experimental evolution may also need to be considered. Although different in many regards from the current study, the work of Perfeito *et al.* [14], for instance, showing a high rate of beneficial mutations in *E. coli* also utilized populations that appear to have experienced daily periods of stationary phase survival between controlled population bottlenecks. If GASP mutants were to arise and reach measurable frequencies (>1%) during a single growth cycle, the probability of adaptive mutations escaping stochastic loss may have been substantially higher than was estimated from fitness values based on changes in microsatellite allele frequencies that were observed once mutants had already risen to measurable levels. Furthermore, for the initial stages of selection when a GASP mutant is at low frequency and may be strongly selected within a single growth cycle, N_e_ may approach the maximum size of the stationary phase population. For events within a single growth cycle, therefore, N_e_ may be similar even for populations subjected to very different bottlenecks. The bottleneck size, however, may still affect the outcome of stationary phase selection because a smaller bottleneck would also correspond to a greater dilution factor and a shorter duration of stationary phase accompanying each growth cycle. In contrast to the rapid initial enrichment of a GASP mutant, the final stages of selection—when a mutant is at higher frequency and less strongly selected—should extend over multiple growth cycles and thereby operate under a lower N_e_ as driven by the recurrent bottlenecks. These considerations may impact the apparent rate of beneficial mutation in studies of adaptive evolution.

Our data imply that either the rate of beneficial mutations in *S. pneumoniae* is quite high (approximately 4.8×10^−4^ per genome per 40 min time interval) or that strong selection is occurring in this system despite a classical mutation accumulation experimental design. The apparent rate of beneficial mutations calculated under our model incorporating weak selection is several orders of magnitude higher than early estimates for bacteria and viruses that clustered near rates of 10^−8^ to 10^−9^ [11], [12], [44]. This estimate, however, more closely agrees with the recent work of Perfeito *et al.* [14] that obtained a beneficial mutation rate for *E. coli* of 2×10^−5^ and for which Monte Carlo simulations of the same data indicated that the actual rate could be as high as 10^−4^. Our findings may therefore provide additional support for the presence of high rates of beneficial mutations among bacteria. The frequency with which such beneficial mutations occur is likely to contribute to the ability of these bacteria to evolve rapidly as conditions change. In the case of *S. pneumoniae*, the spread of these new beneficial mutations may be facilitated by the natural competence of the organism for transformation and may be an important factor in the virulence of this pathogen and in its development of resistance to antibiotics.

## Materials and Methods

### Bacterial Strains and Mutagenesis

Experiments were performed using the encapsulated serotype 2 strain D39 [19] of *S. pneumoniae*. A competence-deficient and kanamycin-resistant mutant, TMP1, was constructed by allelic replacement mutagenesis inserting the *aph3* gene into the *comAB* locus. DNA from a region on the 5′ side of *comAB* was amplified by PCR using the primers 5′-TTTTGTTTAGTGATTGGGGTAAG-3′ and 5′-ACGAGGATCCGAGAGCAGACCATTTTTTTGTTC-3′, and DNA from a 3′ region was amplified with primers 5′-AGCAGGGCCCAATACCAAGAAGGGGCAGAGGG-3′ and 5′-TAGCGAACAGAATCACCGAC-3′. A PCR product encoding *aph3* was amplified from CP1296 DNA [45] using primers 5′-GATCGGATCCGTTTGATTTTTAATGGATAATG-3′ and 5′- ACCTGGGCCCCTCTCCTGTGTTTTTTTATTTTTGG-3′. These products were linked using PCR ligation mutagenesis [46] and introduced into D39 by transformation as previously described [47]. The resulting mutation in *comAB* was confirmed by PCR and sequencing.

### Serial Transfer of Pneumococcal Lines

Serial transfer lines of *S. pneumoniae* were derived from one colony each of strains D39 and TMP1. These colonies were streaked onto fresh THY agar plates (Todd-Hewitt agar supplemented with 0.2% yeast extract), and 20 of the resulting colonies from each (designated growth cycle 0) were picked to initiate the experimental lines. A single colony from each line was chosen daily at random for transfer to a fresh THY plate by selecting the most well-separated colony present on an initial field of view under a stereomicroscope. The interval between inoculation and the next transfer step was maintained between 22 to 26 h and was generally close to 24 h. Between transfer steps, plates were kept at 37°C in an incubator containing ambient air. Samples were preserved at fixed intervals by suspending patches of colonies from the plates in a 4∶1 mixture of THY broth:80% glycerol and stored at −75°C for later testing. During serial transfer, THY plates were divided into 6 radial sectors, which were used for propagation of separate bacterial lines in an alternating pattern such that D39 (kanamycin-sensitive) and TMP1 (kanamycin-resistant) lines were present on adjacent sectors. To check for contamination between lines, we verified that each line had maintained its initial pattern of kanamycin susceptibility or resistance at the end of the experiment.

### Fitness Assays

The growth of isolates on THY agar plates was assessed by dissecting individual colonies under a stereomicroscope after intervals ranging from 7 to 26 h following inoculation. Because we were not able to initiate a single bacterial colony from a controlled mixture of experimental and reference strains, growth was measured by examining colonies composed of a single strain type rather than through direct competition. Six sectors per plate were inoculated with isolates in a randomized order in which passaged and control samples were intermixed. Both the passaged and control isolates had been frozen prior to testing and were grown for 24 h on an initial agar plate before inoculating the colonies to be dissected. The most well-separated colony on a sector was dissected and the number of CFU within the colony was determined by dilution and colony counting. The log_2_(CFU/colony) was used as a measure of the net growth rate of a colony. The ratio of this value for a given isolate to the average value for control isolates from growth cycle 1 (processed in parallel for each assay) was taken as a proxy for fitness. Where a fitness value is given to represent the average of multiple passaged lines, the fitness of each individual line was first estimated by replicate measurements. The average fitness across the lines was then determined as the mean of these estimates for the independent lines, and standard deviation was calculated using a sample size equal to the number of lines.

Growth in liquid culture was measured by suspending individual bacterial colonies in 1 ml aliquots of THY broth. Aliquots were placed in a randomized order into wells of a 48-well plate, which was sealed with optically-transparent film to limit evaporation and incubated at 37°C in a Synergy2 plate reader (BioTek; Winooski, VT). Optical density (OD) readings at 620 nm were taken every 10 min, and the maximum growth rate was estimated by determining the maximum change in log_10_(OD) over any 30 min window. Fitness for growth in this context was calculated as the ratio of the maximum growth rate for a given isolate to the average maximum growth rate for control isolates from growth cycle 1 that were processed in parallel for each assay. For the time series analysis of samples archived at 4-day intervals (Figure 3B), each isolate was compared directly to samples from the preceding time point that were assayed in parallel in order to estimate the change in fitness over the interval (for both broth and agar plate assays) rather than comparing each to the growth cycle 1 reference.

### Maximum Likelihood Model

A computational model was developed to simulate changes in fitness due to mutation and selection, as well as the drift imposed by single-colony bottlenecks, over repeated growth cycles of the serial transfer process. A description of the model is here followed by a more detailed consideration of the assumptions made in the design of the model. The fitness of the population was modeled as consisting of a discrete distribution of fitness classes. Each simulation was initiated with a uniform population having fitness of 1. The model divided each growth cycle into discrete time intervals corresponding to the generation time of *S. pneumoniae* during exponential growth (approximately 40 min in THY medium either on plates or in broth). For simplicity, the first half of the growth cycle was considered to constitute the period of exponential growth while the second half was considered stationary phase.

Beneficial and deleterious mutations were allowed to occur at each time step at rates *U*_b_ and *U*_d_, respectively. The fitness effects of beneficial mutations were assumed to be exponentially distributed with a scale parameter s_b_. Deleterious mutations, however, were considered to have a constant fitness effect s_d_. Fitness was assumed to be multiplicative without epistatic effects. For simplicity, the model did not consider the effects of selection during the exponential phase of growth because our experimental data had indicated that isolates resulting from serial transfer did not have an advantage during this period. Furthermore, the smaller populations present during much of exponential growth would have limited the efficacy of selection during this phase. Mutations therefore were allowed to accumulate neutrally during exponential phase.

Selection during stationary phase operated at the end of each specified time interval and changed the frequency, 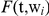, of the *i*th fitness class at time t in proportion to its fitness, w*_i_*, relative to the average fitness of the overall population, 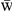, such that:
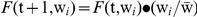
To limit complexity, the variable fluctuations in population size seen in our experimental samples during stationary phase survival were not incorporated into the model.

At the end of each simulated growth cycle, the change in fitness of the bacterium chosen to continue the serial transfer process was determined by a random draw from the fitness distribution after the last time interval. The change in fitness of a bacterial line over the serial transfer experiment was simulated by repeated iterations of the growth cycle model. Because fitness effects were assumed not to show epistasis, the same distribution of fitness changes was used at the end of each growth cycle. Simulation of serial transfer for 10^5^ independent lines was used to generate a distribution of expected fitness values for passaged isolates. This distribution was modified by application of a Gaussian smoothing function with a variance matching the average within-line variance of our experimental data to account for the effects of environmental noise in laboratory measurements of fitness. The log likelihood of the experimental data under the parameters *U*_b_ and s_b_ of a given simulation was calculated as:
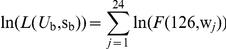
where *F*(126,w*_j_*) is the simulated frequency after 126 growth cycles of the fitness class w*_j_* matching the average measured fitness of the *j*th experimental line of *S. pneumoniae*. Maximum likelihood estimates were then generated for *U*_b_ and s_b_ by exploring the parameter space between *U*_b_ of 1×10^−6^ and 8×10^−3^ and between s_b_ of 0.005 and 0.08. Boundaries of 95% confidence regions were calculated as contours for which the likelihood estimate
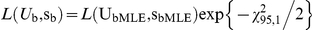
where *U*_bMLE_ and s_bMLE_ represent the ML estimates of *U*_b_ and s_b_, respectively [48].

Although controversy exists regarding mutation rates during stationary phase and the extent to which mutations depend on DNA replication, we assumed initially that mutation rates remained constant as a function of time throughout the growth cycle. The impact of this assumption was tested through a variation in model parameters that eliminated mutations during stationary phase. The potential for selection to amplify preferentially beneficial mutations that have larger fitness effects than average made it important to consider the distribution of effect strengths for these mutations. The actual distribution of selection coefficients for newly arising mutations is uncertain, but the exponential distribution employed here has been proposed on theoretical grounds [48], [49], and the distributions of new beneficial mutations escaping stochastic loss [14] or reaching fixation [48] in *E. coli* show a good match to predictions based on this theory. Recent results also support an exponential distribution for newly arising beneficial mutations in *Pseudomonas fluorescens* [50]. Because the frequency of deleterious mutations causing larger fitness effects than average would be reduced rather than amplified by selection, however, deleterious mutations were considered to have a constant fitness effect s_d_.

Within each growth cycle, the evolution of the bacterial population was modeled deterministically for the reasons outlined below. Because there are approximately 3×10^4^ CFU in each mature colony and there are generally thousands of colonies on a plate at each growth cycle from which a founder may be selected for the next stage, the population from which this founder is chosen is large (at least 10^7^). The overall serial transfer experiment, however, is still characterized by a low effective population size (N_e_ = 18 for the model population) due to the single colony bottlenecks.

In order to estimate the fitness distribution at the end of each growth cycle, rather than modeling the thousands of colonies present during each growth cycle individually we considered these bacteria to represent a single, large population. This assumption reflected the experimental design in which a serial transfer line is propagated through selection of a single CFU from within the collection of colonies present at each growth cycle in a two-step, random process. One colony among many is first randomly chosen for transfer to the next agar plate and spread so as to isolate individual colony-forming units. The random selection of which of these daughter colonies will serve to continue the line is delayed until the end of the next growth cycle, but the evolutionary trajectories of these daughter colonies become independent once they are separated. The model collapses this process into a single random selection event at the end of each growth cycle in which the probability of founding the next population is given by the frequency of a variant within the entire population but is not affected by the distribution of variants among colonies. This strategy reduced the computational complexity of the model by requiring calculation of the fitness distributions only across a single, large, deterministic population rather than in many small populations with stochastic behavior but effectively assumed that bacteria were able to compete equally well across the plate as within a colony. While not strictly realistic, the limited period of stationary-phase selection available before the next growth cycle prevented even highly beneficial mutations from amplifying to a level that would correspond to fixation within a single colony, much less the entire population. (For instance, after 12 hours [i.e., 18 rounds] of stationary-phase selection a new mutation with fitness of 1.5 might be amplified roughly 1500-fold, which would represent less than 5% of a colony having a total size of 3×10^4^ CFU.) The assumption of a well-mixed population did not therefore permit amplification by selection to continue beyond limits compatible with the physical constraint of each variant within a colony.

To simplify the model during the period of neutral mutation accumulation, the frequency of new mutations at the start of stationary-phase selection was given by 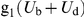, where g_1_ represents the number of time intervals before the onset of selection. Mutations were modeled as if they all occurred in separate individuals (i.e., a Poisson distribution of mutations in the population was not assumed). This simplification was reasonable because the contribution of the exponential distribution of mutational effects toward generating individuals in higher fitness classes is much stronger than the contribution of rare individuals with multiple beneficial mutations. Calculation of the distribution of mutations at the start of stationary phase did not require incorporation of Luria-Delbrück distributions [51] because mutational jackpots within colonies are homogenized among colonies. Because the relative enrichment of mutations within jackpot colonies would have reduced the difference between the fitness of a new variant and that of the colony as a whole, the assumption of a well-mixed population causes the strength of selection to be overestimated in our model. As the frequency of each new variant remains low during a growth cycle (see above), this effect however should be small. Nonetheless, the rate of beneficial mutations inferred using the model may be considered to be a minimum estimate in this regard.

### Stochastic Model of Bacterial Serial Transfer

An alternative and entirely stochastic model was constructed to simulate mutation, selection and replication of individual bacteria within a single propagated colony throughout the serial transfer process. The model was initiated with a single bacterium with fitness of 1. The colony growth cycle was divided into 36 time intervals of equal length, corresponding to the 40 min pneumococcal generation time during exponential growth. At each time interval, individuals acquired beneficial and deleterious mutations with probabilities *U*_b_ and *U*_d_, respectively. Selection coefficients for beneficial mutations were chosen at random from an exponential distribution with scale parameter s_b_. Deleterious mutations were assigned a fixed selection coefficient of s_d_. New mutations had a multiplicative effect on fitness without epistasis. During the first half of the colony growth cycle, each individual replicated at every time interval (i.e., no selection during exponential phase), contributing 2 progeny of identical fitness to the next generation. During the second half of the growth cycle (i.e., stationary phase), acquisition of new mutations continued in the same manner with each time interval. Selection was introduced by stipulating that individuals with fitness less than the population average failed to contribute to the subsequent population with probability
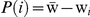
where w*_i_* is the fitness of the *i*th individual and 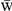 is the mean population fitness. Likewise, individuals with fitness greater than the population average succeeded in replicating during stationary phase (i.e., contributing 2 rather than 1 individuals to the subsequent population) with a probability
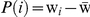
To assess the sensitivity of the model to the choice of fitness function, a variation of the model was also analyzed in which the probability of an individual with below average fitness failing to contribute to the subsequent population was given by
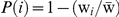
and the probability of an individual with above average fitness reproducing in stationary phase was given by
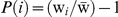
At the end of the growth cycle, a single individual was chosen at random to initiate the next colony of the serial transfer. After iterative modeling of 126 growth cycles, the final fitness for the simulated line was determined by choosing an individual at random at the end of the final growth cycle. Both the stochastic and semi-deterministic models were implemented in Python version 2.6.4 and are available as Text S1.

## Supporting Information

Figure S1 Time series examination of the development of *S. pneumoniae* colonies. Samples were assayed after 7 to 26 h of incubation on THY agar plates. (A and B) Average CFU/colony ± S.E.M. for individual isolates from the 6 serial transfer lines shown in Figure 2 after (A) 1 day or (B) 126 days of propagation. Values for the day 126 isolate from line B are significantly higher than the other lines during stationary phase (*F*_[5,120]_ = 65.76, *P*<0.001). (C) Average CFU/colony ± S.E.M. for 5 of the 6 serial transfer lines shown in Figure 2, excluding line B that showed the most extreme change. The effect of passage on CFU/colony during stationary phase (12 to 26 h) remained significant (*F*_[1,64]_ = 10.86, *P* = 0.0016).
(TIF)

Figure S2 Estimates for *U*_b_ and s_b_ under additional model variations. (A) ML values (triangles) and 95% confidence contours (dotted lines) were derived under the assumptions of (A) deleterious mutations arising at a higher rate *U*_d_ = 5.7×10^−4^ and (B) no new mutations arising during stationary phase. Other model parameters and assumptions were identical to those used for the initial ML estimates shown with the square and solid line in Figure 4B–4D (s_d_ = 0.012, selection throughout stationary phase and, except as noted for S2A, *U*_d_ = 1.7×10^−4^). These initial estimates are shown using the same symbols in both panels here for comparison.
(TIF)

Figure S3 Wholly stochastic simulation of fitness changes during single-colony serial transfer of *S. pneumoniae*. The cumulative distribution of fitness values predicted by the alternative stochastic model as described in the text is shown after 126 growth cycles (diamonds). Fitness values measured for the experimental lines after 126 growth cycles are shown as open squares for comparison. Parameters used for the simulations were *U*_b_ = 4.8×10^−4^, s_b_ = 0.025, *U*_d_ = 1.7×10^−4^, and s_d_ = 0.012. For beneficial mutations, these parameters correspond to the initial ML estimates of *U*_b_ and s_b_ derived from the semi-deterministic model. Results for the stochastic model shown in (A) were generated using selection functions based on differences between individual and average population fitness and in (B) were generated using selection functions based on ratios between individual and average population fitness.
(TIF)

Text S1 Algorithms for semi-deterministic and stochastic models. Python 2.6.4 code for the semi-deterministic and stochastic models is provided as a supplemental text file.
(TXT)
