# Supplementary figures and images for: Frequent Beneficial Mutations during Single-Colony Serial Transfer of Streptococcus pneumoniae
Source: PLoS Genet. 2011 Aug 18;7(8):e1002232. doi: 10.1371/journal.pgen.1002232 (PMC3158050; doi:10.1371/journal.pgen.1002232)

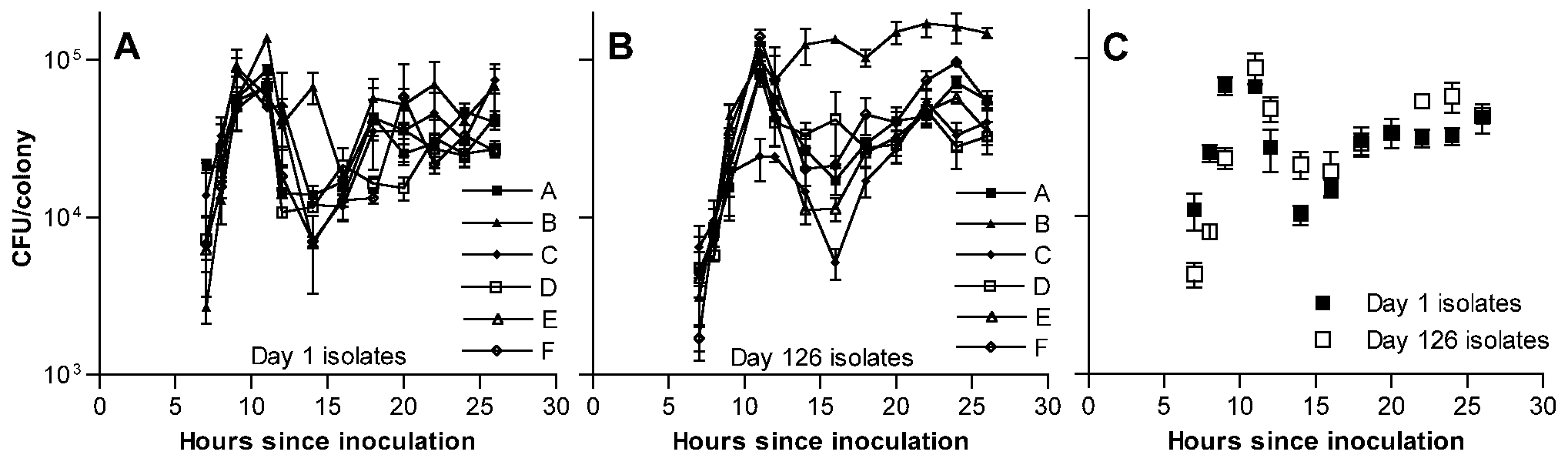

Supplement: Figure S1 — Time series examination of the development of S. pneumoniae colonies. Samples were assayed after 7 to 26 h of incubation on THY agar plates. (A and B) Average CFU/colony ± S.E.M. for individual isolates from the 6 serial transfer lines shown in Figure 2 after (A) 1 day or (B) 126 days of propagation. Values for the day 126 isolate from line B are significantly higher than the other lines during stationary phase (F [5,120] = 65.76, P<0.001). (C) Average CFU/colony ± S.E.M. for 5 of the 6 serial transfer lines shown in Figure 2, excluding line B that showed the most extreme change. The effect of passage on CFU/colony during stationary phase (12 to 26 h) remained significant (F [1,64] = 10.86, P = 0.0016). (TIF) [file pgen.1002232.s001.tif]

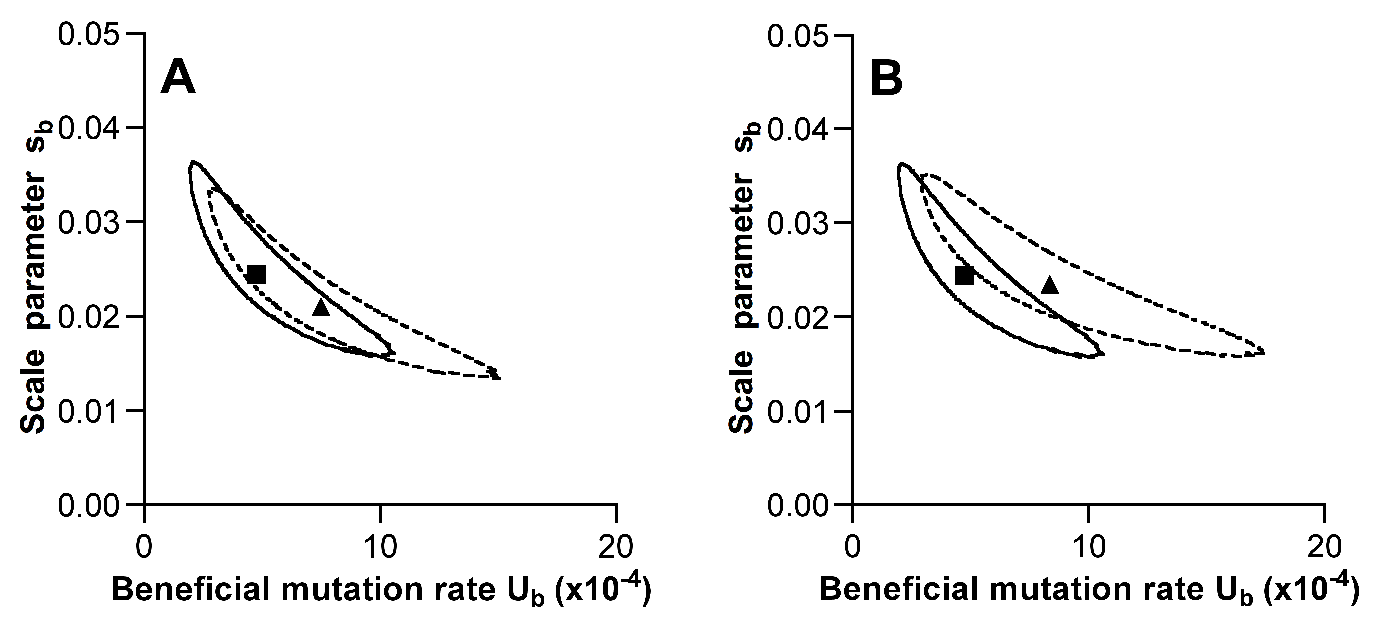

Supplement: Figure S2 — Estimates for U b and sb under additional model variations. (A) ML values (triangles) and 95% confidence contours (dotted lines) were derived under the assumptions of (A) deleterious mutations arising at a higher rate U d = 5.7×10^−4^ and (B) no new mutations arising during stationary phase. Other model parameters and assumptions were identical to those used for the initial ML estimates shown with the square and solid line in Figure 4B–4D (sd = 0.012, selection throughout stationary phase and, except as noted for S2A, U d = 1.7×10^−4^). These initial estimates are shown using the same symbols in both panels here for comparison. (TIF) [file pgen.1002232.s002.tif]

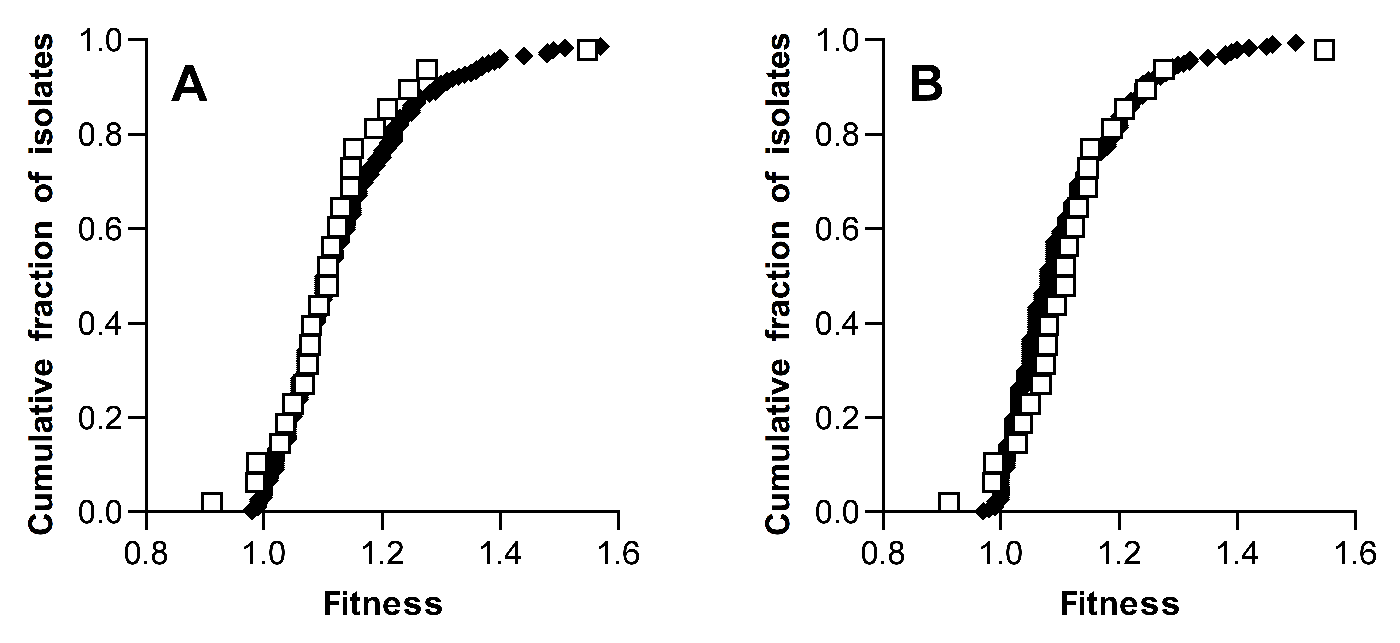

Supplement: Figure S3 — Wholly stochastic simulation of fitness changes during single-colony serial transfer of S. pneumoniae. The cumulative distribution of fitness values predicted by the alternative stochastic model as described in the text is shown after 126 growth cycles (diamonds). Fitness values measured for the experimental lines after 126 growth cycles are shown as open squares for comparison. Parameters used for the simulations were U b = 4.8×10^−4^, sb = 0.025, U d = 1.7×10^−4^, and sd = 0.012. For beneficial mutations, these parameters correspond to the initial ML estimates of U b and sb derived from the semi-deterministic model. Results for the stochastic model shown in (A) were generated using selection functions based on differences between individual and average population fitness and in (B) were generated using selection functions based on ratios between individual and average population fitness. (TIF) [file pgen.1002232.s003.tif]
